# Movement representations in motor, somatosensory, and posterior parietal cortex of the greater galago

**DOI:** 10.1093/cercor/bhaf195

**Published:** 2025-08-21

**Authors:** Andrew C Halley, Iwona Stepniewska, Qimeng Wang, Jamie L Reed, Hui-Xin Qi, Jon H Kaas, Leah A Krubitzer

**Affiliations:** Center for Neuroscience, University of California Davis, Grey Hall, 1544 Newton Court, Davis, CA 95618, United States; Psychological Sciences, Vanderbilt University, 111 21st Avenue South301 Wilson Hall, Nashville, TN 37240, United States; Psychological Sciences, Vanderbilt University, 111 21st Avenue South301 Wilson Hall, Nashville, TN 37240, United States; Department of Radiology, Vanderbilt University, 1211 Medical Center Drive, Nashville, TN 37232, United States; Psychological Sciences, Vanderbilt University, 111 21st Avenue South301 Wilson Hall, Nashville, TN 37240, United States; Psychological Sciences, Vanderbilt University, 111 21st Avenue South301 Wilson Hall, Nashville, TN 37240, United States; Center for Neuroscience, University of California Davis, Grey Hall, 1544 Newton Court, Davis, CA 95618, United States; Department of Psychology, University of California Davis, 135 Young Hall, 1 Shields Ave, Davis, CA 95616, United States

**Keywords:** evolution, primate, prosimian, sensorimotor, stimulation

## Abstract

Previous studies suggest that anterior parietal cortical areas including S1 (3b) play a central role in motor control that is distinct from traditionally defined motor areas of the neocortex (eg M1). The role of anterior parietal areas in generating movement has never been described in any prosimian primate, a lineage thought to reflect the last common ancestor of all living primates. This study describes movements elicited from long-train intracortical microstimulation in the greater galago (*Otolemur garnettii*) in area 3b and adjacent frontal and parietal cortical fields. We found that the representation of forelimb digits was exceptionally small relative to other primate species, while tongue representations were enlarged—possibly an adaptation for frugivory. We discuss these findings in relation to primate behavioral variation, and highlight features of movement representations in 3b and M1 that are common to all mammals studied using similar methods.

## Introduction

Motor control in mammals involves a wide range of cortical areas. While traditionally defined motor areas in the frontal neocortex (eg M1, PM, SMA, etc.) play a central role in motor control of the limbs and body, recent work has shown that parietal regions of the neocortex involved in integrative and sensory functions are also important for motor control. For example, posterior parietal cortex has long been known to play a role in the integration of sensory inputs from the somatosensory and visual systems (Whitlock 2017), and in primates is involved in the generation of coordinate systems (body, shoulder, eye) for reaching and grasping (see Goldring and Krubitzer 2017 for review). Recent work has shown that PPC is also involved in the production of muscle coactivations that produce movement types that are unique to different species, such as manual dexterity in monkeys (Baldwin et al. 2018) or wing orientation during flight in bats (Halley et al. 2022).

Similarly, anterior parietal areas including the primary somatosensory cortex (S1/3b) and areas 1 and 2 were long thought to have a restricted role in processing tactile and proprioceptive inputs from the skin, muscle and joints. However, recent evidence suggests that these cortical areas also play a role in motor control. For example, several studies have shown that anterior parietal areas are central to generating a number of movement types observed within a given species, including S1 in mice (Matyas et al. 2010), rats (Halley et al. 2020), and fruit bats (Halley et al. 2022), and areas 1 and 2 in macaques (Baldwin et al. 2018) and capuchin monkeys (Mayer et al. 2019). Importantly, these studies have shown that stimulation of these areas can evoke movements that are distinct from (and complementary to) those elicited from M1. In mice and rats, stimulation of S1 (3b) evokes whisker retraction and forelimb retraction, while stimulation of M1 evokes whisker protraction and forelimb extension (mice: Matyas et al. 2010; Morandell and Huber 2017; rats: Brown and Teskey 2014; Halley et al. 2020). In fruit bats, stimulation of M1 evokes full extensions of the tongue, while stimulation of 3b evokes tongue retraction or small twitches along the dorsal surface of the tongue (Halley et al. 2022). Further, experiments have shown that inactivation of M1 had a minimal effect on the movements evoked by stimulating area 1 in macaques (Bresee et al. 2024), suggesting parallel pathways for motor control in M1 and regions of anterior parietal cortex.

Taken together, these studies suggest that across mammals, traditionally defined somatosensory areas (3b, 1 and 2) play a role in motor control. Additionally, evidence in some species (rats, mice and fruit bats) indicates that M1 generates suites of muscle coactivations that extend limbs away from the body, while area 3b generates suites of muscle coactivations that retract body parts toward the central body axis (Matyas et al. 2010; Halley et al. 2020; Halley et al. 2022). This distribution of movement types suggests that distinct roles for 3b and M1 in motor control may be a shared feature of the mammalian neocortex, a hypothesis that can only be tested by mapping both fields in additional species.

In the current study we explore the role of anterior parietal areas involved in motor control in the prosimian galago. Galagos are thought to reflect the ancestral form of primates, which were small and nocturnal, subsisted on fruit and insects within an arboreal niche, and in the visual system had a large binocular overlap (Charles-Dominique and Martin 1970; Silcox et al. 2009; Steiper and Seiffert 2012). This supposition is supported by comparative studies of cortical organization in primates and non-primate mammals that indicate that galagos share some features of cortical organization with the closest living relative to primates (tree shrews), but also have distinct features of cortical organization that are common to primates (Kaas et al. 2018; Kaas 2019). Thus, prosimians such as galagos are uniquely positioned for understanding the brain organization of early primates. Previous studies using short-train (ST-) intracortical microstimulation (ICMS) have found a fractured but broadly topographic representation of the body in frontal motor fields (e.g. M1, PM) in both the Brown greater galago, *Otolemur crassicaudatus* (Fogassi et al. 1994) and the species described here, the Northern greater galago, *Otolemur garnettii* (Wu et al. 2000; Stepniewska et al. 2018). More recently, studies have applied stimulation over longer durations (LT-ICMS) and have shown that distinct cortical areas produce different complex movements that involve multiple body parts and joints—suites of movement such as reaching, grasping, hand-to-mouth, forelimb defense and avoidance—both in frontal motor fields (Stepniewska et al. 2020) and in posterior parietal areas (Stepniewska et al. 2005, 2009, 2014; 2020; Cooke et al. 2015). However, previous studies have not examined the role that area 3b plays in motor control in *Otolemur garnettii* or any other species of prosimian.

The current study utilizes long-train intracortical microstimulation (LT-ICMS) in the Northern greater galago to better understand how the primate neocortex generates the variety of motor abilities we observe in natural behavior. Our goal was to examine the types of movements that can be elicited in area 3b, how these evoked movements differ from those elicited in M1, and whether prosimians share features of movement representations found in anthropoid primates, e.g. magnified representations of forelimb digits (Baldwin et al. 2018). Finally, while no living species can represent an ancestral form, prosimian primates like galagos offer a window into the early evolution of the primate branch of mammals, uncovering how the neocortex of our earliest primate ancestors was organized, and the defining features of cortical areas involved in motor control. Equally important, these species can serve as a bridge to understanding how our brains differ from closely related lineages such as rodents—especially mice and rats, two species that serve as the primary animal models used in neurobiology. This study is part of a larger comparative effort in our laboratory using identical stimulation methods to describe how motor organization has evolved in diverse mammals, including primates (Baldwin et al. 2018; Mayer et al. 2019), tree shrews (Baldwin et al. 2017a), rats (Halley et al. 2020), and bats (Halley et al. 2022).

## Materials and methods

Intracortical microstimulation (ICMS) was used to characterize movements elicited from frontal and parietal cortex in four male adult Northern greater galagos (*O. garnettii*). All animals were 3.5–6 years old with an average weight of 1273 g. Experimental procedures were approved by Vanderbilt University IACUC and conform to NIH guidelines.

### Surgical procedures

Anesthesia was induced with an intramuscular injection of ketamine (10–30 mg/kg) and subsequent surgical procedures were carried out under isoflurane anesthesia (2.5%). At the time of induction, intramuscular injections of buprenorphine (0.005 mg/kg), ceftiofur sodium (50 mg/ml; 2.2 mg/kg), glycopyrrolate (0.015 mg/kg), and dexamethasone (1.0 mg/kg) were also given. Following surgery, animals were maintained under anesthesia using intravenous administration of ketamine (10–20 mg/kg/hr) and intramuscular injections of xylazine (0.5–2 mg/kg every 2–4 hours). In three cases, diazepam was administered intravenously to prevent seizures (0.5–1.0 mg/kg) in consultation with a veterinarian. We administered a continuous infusion of lactated Ringer’s solution with 2.5%–5.0% dextrose throughout (1–3 mL/kg/hour; IV). A steady level of anesthesia was maintained by monitoring heart rate, respiratory rate, body temperature, oxygenation levels, muscle tone, and both eye-blink and pinch responses.

**Fig. 1:**
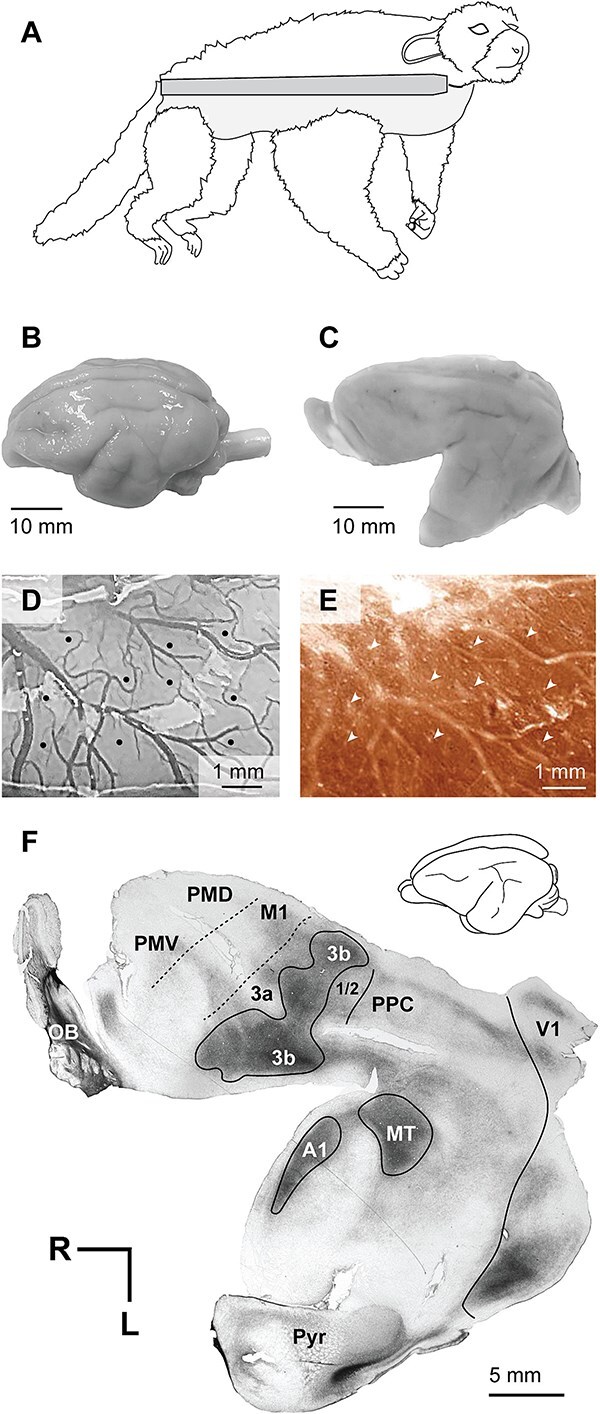
Methods for motor mapping and reconstruction of electrode sites relative to cortical field boundaries. (A) A hammock was used to allow unrestricted movement of the limbs during stimulation, (B) the whole brain was extracted, and (C) the experimental cortical hemisphere was flattened. (D) Stimulation sites (black circles) were noted on a photograph of the brain surface. (E) Electrode penetrations (white arrows) were identified relative to vasculature on sections of the cortical surface stained for cytochrome oxidase. (F) Functional data was then directly related to cortical field boundaries on deeper sections stained for myelin (shown) and CO.

Following anesthetic induction, animals were intubated, cannulated, and catheterized. Lidocaine (2%) was injected subcutaneously behind the ears and along the midline of the scalp, and topical lidocaine ointment was applied to the ear canals. To prevent drying, ophthalmic ointment was applied to the eyes. The skin and temporal muscles were retracted bilaterally to expose the skull. In the right hemisphere, small screws were inserted into the skull and attached to a stereotaxic frame using dental acrylic. In the left hemisphere, a craniotomy was performed to expose a large region of frontal and parietal cortex, after which the dura was retracted and silicone fluid was applied to prevent desiccation. Digital images were taken of the exposed cortex so that stimulation sites could be directly related to cortical vasculature. Following surgery, animals were transferred to a cloth hammock that allowed free movement of the hindlimbs, trunk, forelimbs, and face (see Fig. 1A).

### ICMS mapping

A Master 8 stimulator (AMPI) was used to apply 500 ms long trains of 0.4 ms biphasic pulses, delivered at 300 Hz, to the neocortex via a low-impedance tungsten microelectrode (1.0 M-Ohm). Using a micromanipulator, the electrode was lowered to a depth of 1800 μm at sites perpendicular to the cortical surface, and a depth of 1900–2200 μm at a small number of sites that were oblique to the cortical surface. Each electrode site was recorded relative to vascular patterns on a high-resolution photograph of the cortical surface (Fig. 1D).

**Fig. 2:**
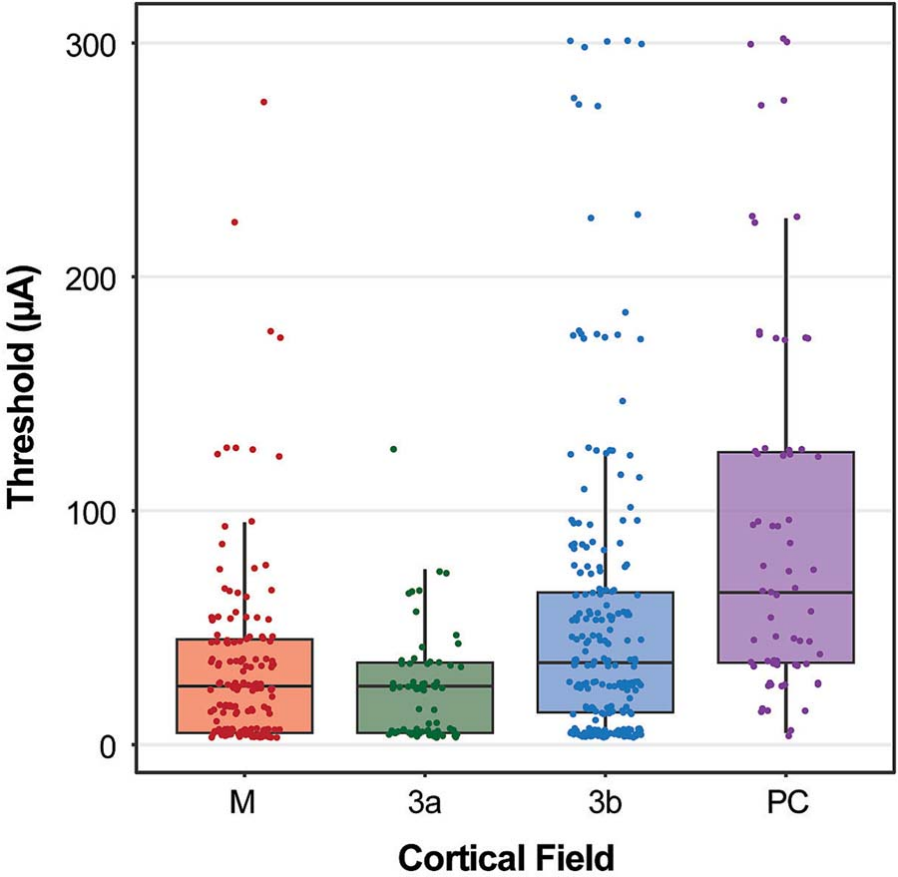
Boxplot of LT-ICMS stimulation thresholds across all four experiments, according to cortical field. Each data point indicates a single stimulation site in an individual case. “M” includes primary motor cortex, as well as premotor regions. “PC” includes areas 1, 2, and PPC (see text). See methods for details.

**Fig. 3:**
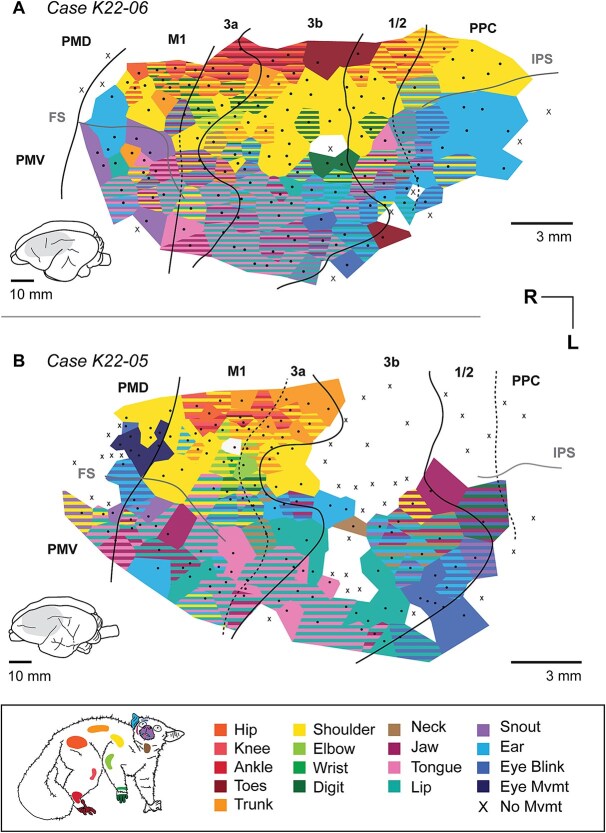
Movement maps in cases (A) K22–06 and (B) K22–05. Using long-train (LT-) ICMS, movements were elicited from premotor, motor, somatosensory, and posterior parietal areas of the neocortex. While movement maps were fractured, there was a gross somatotopic organization of movements in each case—moving from medial to lateral areas of the neocortex, movements were elicited from the hindlimb, the forelimb, and the face. Black dots indicate electrode sites, and surrounding polygons are colored to indicate movements elicited from stimulation of that site. Note that at many sites where movements were evoked, multiple body parts were involved in the movement (striped polygons). Polygons that include multiple colors indicate sites for which multiple body movements were elicited at maximum stimulation (300 μA). X’s indicate electrode sites at which no movements were elicited. Solid lines indicate the borders of cortical fields, determined from histological sections stained for myelin and cytochrome oxidase. Dotted lines indicate borders that were unclear from myeloarchitecture, and were estimated from previous studies. Simplified versions of these maps are provided in Fig. S3.

**Fig. 4:**
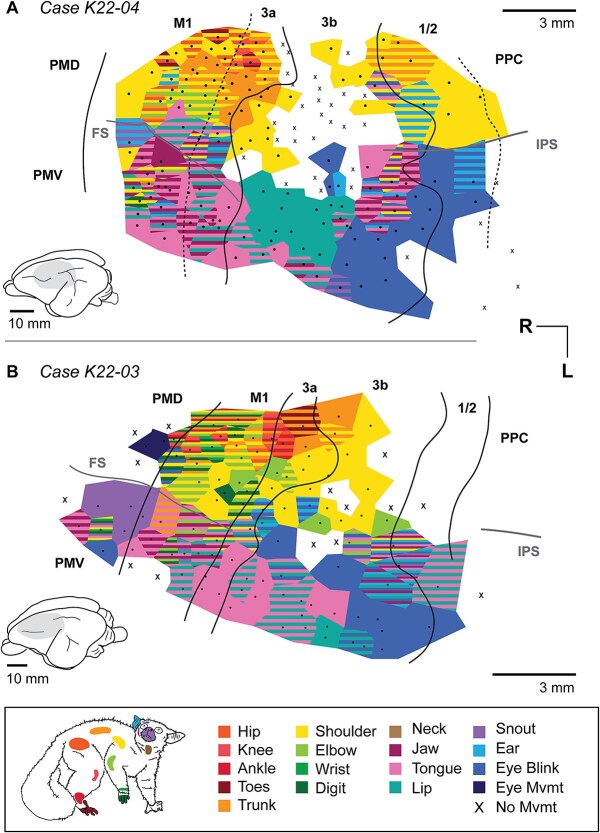
Movement maps in cases K22–04 and K22–03. The overall topographic organization was similar to that observed in the previous two cases. However, there are some notable differences. First, there is a relatively large region of cortex in which the lips (A) or tongue (B) are represented individually rather than in conjunction with other face movements. Second, there is a relatively large area in which eyeblinks could be evoked. Also of note was that in one case (A), fewer sites evoked movements in S1. Conventions follow those of Fig. 3. Simplified versions of these maps are provided in Fig. S2.

Elicited movements were confirmed by two observers and recorded in a surgical log, and video recorded (GoPro Hero 7; resolution 1920 × 1440) relative to a scale bar for offline frame analysis. Lens distortion was removed using Adobe Premier Pro prior to frame analysis. Each stimulation train was synchronized to both a speaker (audible to observers) and an LED placed within the recording frame. Movement thresholds were measured by taking the average between (i) the lowest current at which a movement was observed, and (ii) the next-lowest current at which that movement was not observed. The interval between these measurements was 10 μA for thresholds below 150 μA, and 50 μA for thresholds above 150 μA. Figure 2 shows a boxplot of thresholds observed in each cortical field across all four cases. Movement maps (Figs. 3 and 4) show suprathreshold movements, as in previous studies from our laboratory (e.g. Halley et al. 2022, 2020; Cooke et al. 2012; Baldwin et al. 2017a, 2018). At each site, we noted which body part movements were evoked during stimulation (e.g. elbow, wrist, digits) and the type of movement evoked (e.g. retraction, extension, pronation).

### Histological processing

At the end of each experiment, animals were euthanized with an intravenous injection of pentobarbital sodium and phenytoin sodium (Euthasol, > 120 mg/kg, IV), then perfused transcardially with phosphate buffered saline (0.9%) followed by 2% paraformaldehyde, and finally 2% paraformaldehyde with 10% sucrose. Immediately following extraction (Fig. 1B), the experimental hemisphere was separated from the thalamus and brainstem, and the cortex was manually flattened (Fig. 1C). Flattened hemispheres were post-fixed in 4% paraformaldehyde for 2–7 hours, and then placed in 30% sucrose phosphate buffered solution for 48–96 hours for cryoprotection prior to sectioning on a freezing microtome.

Each flattened hemisphere was cut tangential to the cortical surface at a thickness of 50–60μm. The first two to four sections were stained for cytochrome oxidase (CO) which revealed the vascular patterns of the exposed cortex (Fig. 1D, E); subsequent sections were stained for CO and myelin in an alternating series to reveal cortical field boundaries (Fig. 1F) as described by Wong and Kaas (2010). Abbreviations of cortical fields, brain areas, and sulci are listed in Table 1. High-resolution scans of stained tissue were taken using a Nikon Multiphot (Tokyo, Japan) with a Phase One Powerphase FX1 scan back (Global Manufacturing, Louisville, CO) at the Center for Neuroscience at UC Davis. Scanned photos were adjusted for brightness and contrast using Adobe Photoshop.

**Table 1:** Abbreviations of cortical fields, brain areas, and major sulci.

1/2	Areas 1 and 2	IPS	intraparietal sulcus	OB	Olfactory bulb
3a	Area 3a	LS	Lateral sulcus	PPC	Posterior parietal cortex
3b	Area 3b (S1)	M1	Primary motor cortex	Pyr	Pyriform cortex
A1	Primary auditory cortex	MT	Middle temporal area	V1	Primary visual cortex
FS	Frontal sulcus	PM	Premotor cortex		

### Aligning functional data with cortical boundaries from histology

Electrode sites were identified relative to cortical field boundaries on stained sections by comparing the vasculature seen in photographs of the cortical surface (Fig. 1D) with superficial sections of the flattened cortex stained for CO (Fig. 1E). Cortical field boundaries were determined from deeper sections of flattened cortex, registered to the surface section. Movement maps (Figs. 3 and 4) were constructed from this combination of functional and histological data. Map figures were generated in Adobe Illustrator; the polygons that surround each electrode site were generated using a Voronoi tessellation script (https://github.com/ff6347/Illustrator-Javascript-Voronoi). Polygons filled with a single color indicate sites that produced a single body movement, while polygons that are tiled with multiple colors indicate sites that produced multiple body part movements, corresponding to the colors in the polygon.

### Averaging functional data

**Fig. 5:**
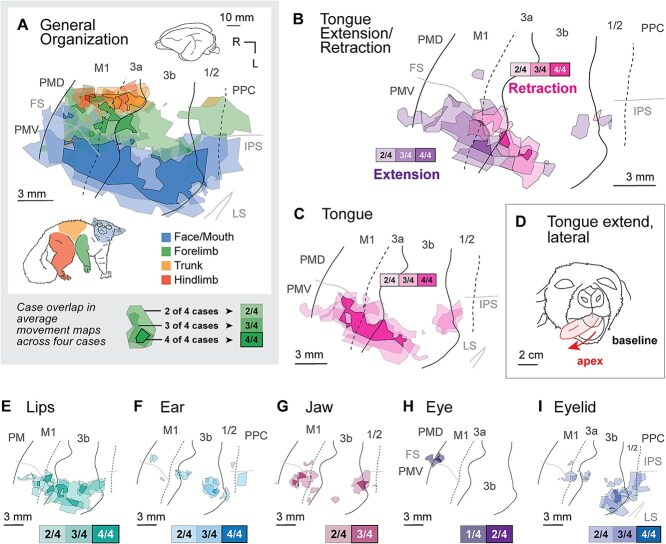
Overlap in the movement representations across four cases relative to averaged cortical field boundaries and sulci (see Methods). (A) A summary of movements observed from four major body parts across all cases, showing a rough somatotopic organization across cortical fields, with hindlimb movements represented medially and face/mouth movements represented laterally. (B–I) Movements of the tongue, lips, ear, jaw, eye, and eyelid. (B) Stimulation sites that evoked tongue retractions are concentrated in medial and caudal portions of the tongue representation, and found mainly in 3b and 3a. Sites that evoked tongue extensions are located rostrolaterally and are concentrated in 3b, 3a, M1, and PMV. (C) The location of tongue movements of all types. The outline indicates all head/face movements. (D) An example of tongue extension and lateral movement, with the apex of movement shown in red. (E) Lip movements were evoked across a wide portion of the face representation (see details in fig. S5B). (F) Ear movements were evoked in medial portions of the face representation in 3b. (G) Jaw movements were evoked in a rostral area (3a, M1) as well as an area of caudal 3b and 1/2 (see details in fig. S5A). (H) Eye movements were elicited from the frontal eye field. (I) Eyelid movements were elicited from a region of 3a adjacent to the FEF, as well as a caudal region of 3b and adjacent area 1/2. Scale bars = 3 mm in (E–I).

**Fig. 6:**
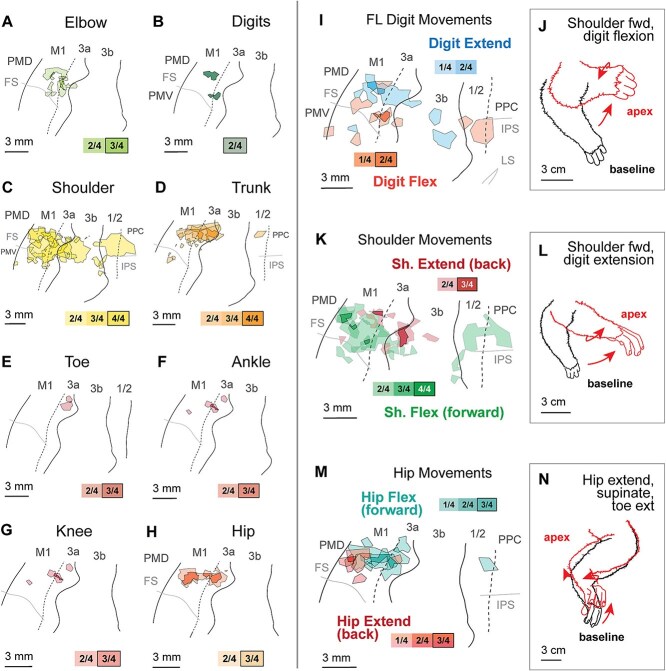
Average limb movements across all cases, following conventions in Fig. 5. Most sites in which movements of the forelimb were evoked were medial to the face representation (A, B) Elbow and digit movements were evoked mainly in areas 3a and M1. (C) Sites in which shoulder movements were evoked were broadly distributed across all cortical fields examined and were the most common movement in the forelimb representation. (D) Trunk movements were evoked in rostral S1, 3a, and M1. (E–H) Sites which evoked movements of the toe, ankle, knee, and hip were concentrated in areas 3a and M1. (I) Distribution of forelimb digit movement types that were evoked, with an example of digit flexion coupled with shoulder flexion in (J). Distribution of stimulation sites that evoked different directions of (K) shoulder movements with an example of forward motion in (L). (M) Distribution of sites that evoked hip movement in different directions, with an example of forward extension in (N).

In order to produce an average organization of the movement types elicited from stimulation across the four individual cases, functional datasets were aligned using a combination of cortical field boundaries (e.g. rostral border of area 3b) and morphological landmarks (e.g. the frontal sulcus). Following this alignment, average cortical field boundaries and major sulci were generated by averaging vectors of their location in Adobe Illustrator. Functional data was averaged across cases by overlapping the polygons corresponding to movements of a given body region (e.g. shoulder, tongue), or a given directional movement of that body part (e.g. tongue extension vs. retraction). This generates average polygon regions for each given movement across the cases, where darker color shades indicate regions in which a given movement was observed in a larger number of cases. In each averaged map, the shade of a given color corresponds to the number of overlapping cases, shown in a key (e.g. “2/4” indicates that in two of the four cases, movements were elicited at that location). This method has been described in detail previously (Halley et al. 2020). Average maps of the face and head are shown in Fig. 5, and maps of the forelimb and hindlimb are shown in Fig. 6.

### Data analysis

The minimal current at which movements could be elicited (i.e. thresholds) were recorded at each stimulation site. For each case and for the total dataset, Table 2 describes summary statistics of the total number of stimulation sites, number of sites at which movements could be elicited, sites with threshold data, and average thresholds. Figure S3 shows the averaged thresholds across all cases relative to averaged cortical fields. The surface area of tongue, shoulder, and forelimb digit representations were measured (mm^2^) in Adobe Photoshop using the scale of individual cases and measuring the size of polygons including those movements. These individual body part representations are compared against the surface area of the entire excitable cortex in each case, and are presented as percentages (Table 3).

**Table 2:** Summary of site numbers and average thresholds across cortical fields in four experiments. Site numbers include the total number of electrode penetration sites, the number of sites at which movements could be elicited, and the number of sites at which thresholds were measured. Movement threshold sites are then presented by cortical area location. Motor cortex includes both primary motor cortex (M1) and premotor cortex (PM). Posterior cortex includes areas 1 and 2 and posterior parietal cortex (PPC). These regions were grouped because boundaries were indistinct in flattened preparations.

	*All Cases*		*K22–03*		*K22–04*		*K22–05*		*K22–06*	
	*Site #*	*Th (**μ**A)*	*Site #*	*Th (**μ**A)*	*Site #*	*Th (**μ**A)*	*Site #*	*Th (**μ**A)*	*Site #*	*Th (**μ**A)*
Total Sites	633	n/a	113	n/a	175	n/a	176	n/a	169	n/a
Mvmt Sites	541	n/a	101	n/a	142	n/a	140	n/a	158	n/a
Thresh Sites	521	45.6	97	37.3	138	57.7	133	54.4	153	32.2
PM/M1	185	32.0	43	20.4	49	31.9	64	45.9	29	20.9
3a	71	20.3	14	10.4	23	31.7	15	25.0	19	8.8
3b	220	55.1	40	59.7	59	74.7	58	68.4	63	21.9
1/2/PPC	65	79.7	4	102.5	11	140.0	3	108.3	47	62.3

**Table 3:** The size of movement representations in absolute surface area (mm^2^) and as a percentage of all movements elicited (“All”) for shoulder, digit, and tongue. Representations of the tongue are exceptionally large (29.5%), while representations of the digits are exceptionally small (5.3%), especially relative to shoulder movements (35.0%). Sites were included if movements were observed up to 300μA, as in primary figures (see Methods). The averages in the “all cases” column are the simple mean of percentages for the four cases.

		*All Cases*	*K22–03*		*K22–04*		*K22–05*		*K22–06*	
		*%*	*mm^2^*	*%*	*mm^2^*	*%*	*mm^2^*	*%*	*mm^2^*	*%*
All		n/a	81.6	n/a	117.5	n/a	128.6	n/a	187.8	n/a
Shoulder		35.0%	26.3	32.2%	47.2	40.1%	34.6	26.9%	76.5	40.8%
Digit		5.3%	7.6	9.3%	0.4	0.4%	6.0	4.7%	12.5	6.6%
Tongue		29.5%	30.9	37.8%	34.5	29.3	31.5	24.5%	49.3	26.3%

## Results

Movements of the hindlimb, trunk, forelimb, and face were elicited from 541 total sites across four cases in cortical areas M1, PM, 3a, 3b, 1/2, and PPC (Figs. 3 and 4; Table 2). We found the lowest stimulation thresholds were observed in PM/M1 as well as area 3a, while the highest thresholds were observed in areas 1/2/PPC (Fig. 2; Table 2).

A range of distinct movements were evoked for multiple body regions following stimulation of these cortical areas. In the following results we first briefly describe the myeloarchitecture of cortical fields in which movements were evoked. We then describe the overall organization of movement maps from the four cases we examined, which are illustrated in Figs. 3 and 4. Finally we describe an “average” or composite map generated by combining the four cases anatomically (by body part) and by movement type (e.g. extension vs. retraction) and illustrated movement examples for these maps (Figs. 5 and 6).

### Cortical architecture

The architectonic appearance of motor and somatosensory cortex in galagos has been described previously utilizing different histological markers in different planes of section (Wong and Kaas 2010). Here we briefly describe cortical areas from which movements can be evoked in cortex that has been flattened and cut parallel to the cortical surface and then stained for myelin. When defining architectonic boundaries, the entire series of sections is used, as individual sections may not show all of the boundaries (Fig. 1F). The compilation of individual sections and their boundaries revealed a number of distinct cortical fields. First, area 3b is distinguished as a very densely staining cortical field which is irregularly shaped, and in some locations separated by lightly myelinated strips of cortex. Area 3a appears as a moderately myelinated strip of cortex at the rostral boundary of area 3b. Area 1/2 is a moderately myelinated strip at the 3b caudal boundary. M1, located just rostral to area 3a, is lightly myelinated and in most cases, the full boundary of M1 was obtained across multiple sections. PPC is lightly to moderately myelinated, allowing us to readily draw its boundary with area 1/2 in some cases (Fig. 3A, 4B). We were unable to distinguish the caudal boundary of PPC accurately from our preparations.

As noted above, our methods allowed us to align our architectonic data directly with our intracortical microstimulation maps so that individual stimulation sites could be accurately assigned to a given cortical field. When area borders were difficult to distinguish, dashed lines were used to mark boundaries based on other cases in this study, as well as previous studies (e.g. Wong and Kaas 2010).

### Movement map organization in areas M1, 3a, 3b, and PPC

In each of the four cases we were able to evoke movements in a large swath of cortex (~15–22 mm of cortex along the rostrocaudal axis, and 9–12 mm of cortex along the mediolateral axis). A range of distinct movements were evoked for multiple body parts following stimulation of M1/PM (185/541 sites), 3a (71/541 sites), 3b (220/541 sites) and area 1/2 and PPC (65/541 sites). Unlike sensory maps, movement maps were not tightly organized topographically, but rather we observed fractured maps in which individual stimulation sites evoked movements of multiple body parts. Thus, although a gross topography can be identified across cases in which the foot and hindlimb are represented most medially followed by the representations of the forelimb and face laterally (Fig. 5A), there was a good deal of variability between cases. Further, while different cortical fields could generally be distinguished architectonically, determining their functional boundaries was often difficult for two reasons. First, low thresholds were found not only in primary motor cortex (M1), but also adjacent fields 3a and 3b, and threshold level varied within each of these fields (Table 2; Fig. S5). Second, individual fields did not contain a complete representation of movement types, but rather some movement types were restricted to a particular cortical field.

There were several noteworthy observations from our study which will be described in detail below. One of the most interesting results was our ability to evoke movements in area 3b and area 1/2, which has not been reported in previous studies of galagos. Other important results included the magnification of particular body part representations such as the tongue and shoulder, the coactivation of body parts involved in what appeared to be ethologically relevant movements, bilateral activation of some body parts, and a conspicuous lack of sites in which stimulation evoked movements of digits alone. In addition, movement types for some body parts were not represented continuously across all the fields examined but were in distinct locations in some, but not in all of the cortical fields. Finally, while we did encounter stimulation from which movement could be evoked from a single joint, most stimulation sites evoked movements from several body parts that act synergistically, similar to previous studies in galagos (e.g. Stepniewska et al. 2005, 2009).

### Face and mouth movements

Movements of the face and mouth were elicited from stimulation of a continuous region that spans PM, M1, 3a, 3b, 1/2, and PPC, representing the tongue, lips, ear, jaw, eye, and eyelid (Fig. 5). One of the distinguishing features of the movement maps of the face was the large magnification of tongue movements, which were observed in areas M1/PM, 3a, 3b, and to a lesser extent area 1/2. These movement representations comprised on average 29.5% (range 24.5% to 37.8%) of the sites from which movements could be evoked. (Table 3; Figs. 3, 4). Tongue extensions were evoked from stimulation of sites in lateral portions of M1 and 3a, while tongue retractions were evoked from sites in more medial areas of sensorimotor cortex, including areas 3a and 3b (Fig. 5B). Lateral tongue movements and repetitive movements of the tongue were elicited from PMD, M1, and 3b (Fig. S5D). Upward and downward tongue movements were distributed across every cortical area (Fig. S5C). An example of tongue lateral tongue extension is shown in Fig. 5D.

Lip movements were also elicited from a large portion of every cortical field, and in two cases (Fig. 3) almost always overlapped with tongue regions (Fig. 3A and B). While coactivation of the lips and tongue were observed in the other two cases, there were also large regions of cortex in which stimulation evoked movements of the tongue and lips in isolation (Fig. 4A and B). Sites in which movements of the ear, jaw, and eyelid were elicited (Fig. 5F, G, I) were not continuous across the five fields in which we could evoke movements, but were concentrated in different cortical areas: ear movements were mostly evoked caudally in area 3b; jaw movements were evoked at the M1/3a boundary and in area 1/2; eyelid movements (blinks) were evoked predominantly in caudal area 3b and area 1/2, and to a lesser extent in area 3a. As shown in the supplementary figures, evoked jaw openings and closings were mostly non-overlapping and concentrated in different cortical fields. Detailed movement maps of the jaw and lip are shown in Fig. S5A, B.

### Forelimb movements

Forelimb movements were elicited from stimulation of every cortical area investigated (Fig. 5A), and most of the forelimb region was occupied by the representation of the shoulder (35% of total movement sites; Table 3; Fig. 6C). Sites in which stimulation evoked movements of the elbow and digits were relatively small and were concentrated in areas 3a and M1 (Figs. 4A, B). Unlike New World and Old World monkeys in which similar methods were used to explore cortical areas involved in motor control (Baldwin et al. 2018; Mayer et al. 2019), sites at which stimulation evoked movements of the digits alone were relatively rare (Figs. 3 and 4). Representations of the digits made up a small proportion of total maps (5.3%; Table 3). In most cases, movements of the digits were coupled with movements of the shoulder and elbow.

We categorized shoulder movements into two major types, extension and flexion. Shoulder extensions—moving the entire forelimb backward—were concentrated in rostral areas 3b, 3a, and M1, while shoulder flexions—moving the forelimb forward—were concentrated in two regions: a rostrolateral portion of 3a and M1, as well as a caudal region in areas 1/2 and PPC (Fig. 6K). These patterns match those for complex forelimb movement types, with shoulder flexion vs. extension corresponding to “raise” vs “retract” movements (Fig. S6A) respectively. Elbow movements were elicited from stimulation of area 3a and M1 in all cases (Fig. 6A), and from stimulation of rostral area 3b in one case (K22–03). Movements of individual digits were primarily elicited from stimulation of M1, but were also observed in areas 3b, 3a, and 1/2 (see Fig. 3; K22–06, K22–05), most often in combination with other parts of the forelimb. These movements of digits were relatively rare, and make up an average of only 5.3% of the excitable area of cortex across the four cases. Forelimb digit representations are much larger in some species of Haplorrhines (see Discussion).

### Hindlimb & trunk movements

Hindlimb movements were primarily elicited from stimulation of sites in areas M1, 3a, and the rostral portion of area 3b (Fig. 5A). In one case with extensive 3b representations (Fig. 3A), toe movements were elicited from most of medial area 3b. Movements of the distal hindlimb (knee, ankle, toe) were concentrated in area 3a (Fig. 6E-G) and adjacent fields. Movements of the hip overlapped with this area, but also extended rostrally into M1 (Fig. 6H). Hip flexions were concentrated in M1 and 3a, while hip extensions were largely observed in regions immediately rostrolateral, in M1, and caudal, in 3b and 1/2 (Fig. 6M). An example of hip extension, combined with supination and toe extension, is shown in Fig. 6N. Trunk movements were elicited primarily from sites in M1, 3a, and the rostral portion of 3b (Fig. 6D). Additional trunk movements were evoked in motor regions lateral to the frontal sulcus (Fig. 3A;  4A;  4B) and caudal regions along the border of 3b, 1/2, and PPC (Fig. 3A;  3B;  4A).

### Bilateral movements and forelimb/hindlimb synergies

Bilateral movements of the forelimb were primarily elicited from stimulation of areas M1, 3a, and rostral area 3b (Fig. S3A), as well as caudal 3b in some cases (Fig. S3A). Bilateral hindlimb movements were concentrated in the medial portions of M1 and 3a (Fig. S3B). For both forelimb and hindlimb, bilateral movements were elicited preferentially from more rostral portions of each representation.

**Fig. 7:**
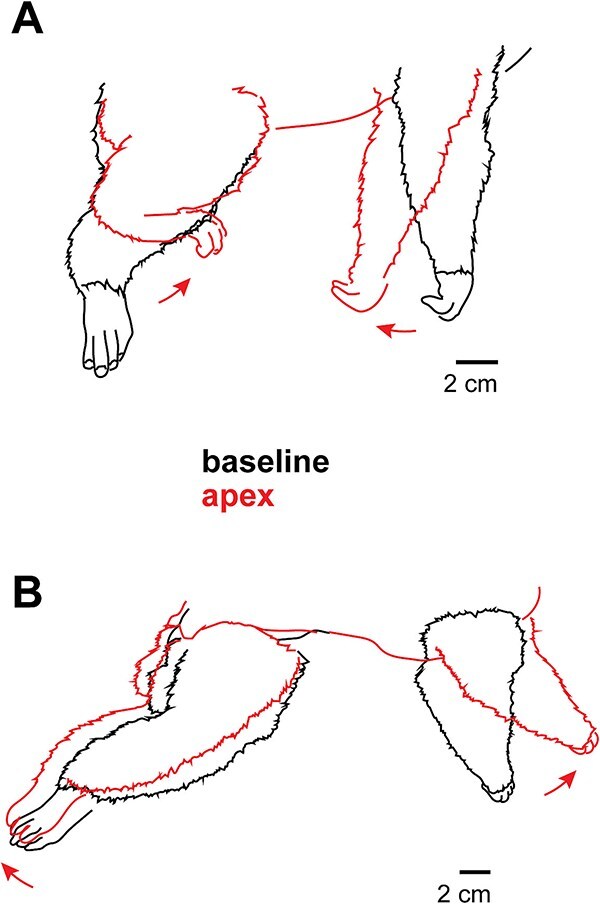
Bilateral coactivation of the forelimb and hindlimb, elicited from two different cases, and in different directions. (A) Stimulation of a site in M1 in case K22–06 caused both limbs to retract toward the midline, involving shoulder extension and hip flexion. (B) Stimulation of a site in S1 in case K22–04 caused both limbs to stretch outward, including shoulder flexion and hip extension. The full extensions observed in (B) closely resemble galago orientation while leaping between branches.

Coordinated movement of both forelimb and hindlimb from a single stimulation site was common, with two basic patterns of coactivation. First, a general retraction of the limbs inward toward the midline of the body, involving shoulder and hip adduction (e.g. Fig*.* 7A, a site in medial M1 in case K22–06). Second, a general forward reach at the shoulder (flexing forward) and extension of the hip backward (Fig. 7B, a site in medial area 3b in case K22–04). These coactivations are similar to the locomotor pattern of clinging-and-leaping observed in natural galago behavior.

In two cases, K22–04 (Fig. 4A) and K22–05 (Fig. 3B), we did not evoke responses from stimulation of the medial portion of area 3b. Stimulation of these same regions elicited movements in K22–06 (Fig. 3A) and to some extent in K22–03 (Fig. 4B). Variation in anesthetic levels across experiments are likely involved in this variation, specifically the administration of ketamine and xylazine, adjusted according to real-time monitoring of vital signs (see Methods). We tried to mitigate the problem of anesthetic variability across the experiment by randomizing site locations (e.g. a site in PPC, followed by a site in M1), rather than testing adjacent cortical sites in sequence. Despite this, we found less consistent responses and higher stimulation thresholds (Fig. S3) in medial 3b. Interestingly, we have observed the same effect in studies using the identical technique in rats (c.f. the lack of medial movement in Halley et al. 2020: Fig. 5) and fruit bats (e.g. movement and threshold variation in Fig. 1D, Fig. S2B from Halley et al. 2022). Briefly, medial portions of 3b—representing the forelimb, torso, and hindlimb—have the highest stimulation thresholds, are the first to disappear as anesthetic levels increase, and are sparse in cases K22–04 (Fig. 4A) and K22–05 (Fig. 3B). In contrast, stimulation of lateral portions of 3b—representing the head and face—produced movement in every case, and at lower thresholds than medial 3b.

We suspect that a major source of differences in responsiveness and threshold variation between medial and lateral 3b is that the former rely on corticospinal tracts, while the latter rely on corticobulbar connections. This methodological issue can have an impact on the interpretation of the results about the relative size of different movement representations across cortical fields. For this reason Table 3 contains both the average relative size of the shoulder, digit and tongue representations as well as the relative size of these representations for individual cases.

## Discussion

This study of the Northern greater galago is the first to describe movement maps in the primary somatosensory cortex (area 3b) and area 1/2 in any species of Strepsirrhine or prosimian primate. In order to appreciate the full extent of cortex from which movements could be evoked, we also characterized the movements elicited from neocortex spanning traditional motor areas (PM, M1), as well as parietal areas adjacent to area 3b and area 1/2 (area 3a, PPC). Like other mammals investigated using similar techniques, we found that galago area 3b plays a role in generating the range of movements observed in natural behavior (e.g. Baldwin et al. 2018; Halley et al. 2022).

A number of anatomical substrates may contribute to the movements elicited from stimulation of parietal cortex. The corticospinal tract has been shown to originate in area 3b, 1/2 and PPC in galagos, other primates and non-primate mammals (e.g. Nudo and Masterton, 1990; Remple et al, 2006; Galea and Darian-Smith 1994; Lemon and Morecraft 2023). Alternative pathways include connections between these areas and motor cortex (Liao et al. 2013; Fang et al. 2005), as well as transthalamic pathways (Sherman 2016).

In terms of overall movement map organization, we observed a relatively small region of the digit representation compared to other primates; an expansion of the tongue representation; and muscle synergies involving the forelimb and hindlimb that are consistent with galago locomotion. While the phenotype of movement maps in motor and parietal cortex in galagos is different from those in other mammals in which similar techniques were utilized in terms of the types of synergies represented (e.g. bats, rats, tree shrews, macaques, capuchins), the general principles of organization are the same: commonly utilized muscle synergies related to ethologically relevant behaviors are magnified in movement maps. Below, we describe these findings in detail and then compare our results with earlier studies of the galago neocortex. Finally, as this study is part of a larger comparative project on the evolution of motor neocortex, we compare our galago findings to other species (both primate and non-primate mammals) that have been studied using similar techniques.

### Muscle synergies for directional movement

A number of studies have suggested that area 3b and M1 play distinct and complementary roles in the generation of directional movement, including protracting vs. retracting the whiskers (Matyas et al. 2010) and extending vs. retracting the forelimb (Halley et al. 2020). In contrast, in the current study we found that the differences in movement for a given body part (e.g. “shoulder flexion vs. extension”) did not often correspond to the boundaries of cortical areas, defined using myeloarchitecture. Instead, movement types were usually distributed across multiple cortical fields (e.g. “tongue retraction” observed along a continuous strip of M1, 3a, 3b, 1/2 [Fig. 5B]), and orthogonal to the boundaries of those fields. Complementary movement types were observed in adjacent regions of this sensorimotor cortex, usually along a a combination of medial-lateral and rostral-caudal axes. Tongue retraction was concentrated medial to tongue extension (Fig. 5B). Forelimb digit extension was concentrated medial and caudal to digit flexion (Fig. 6I). Shoulder extension was concentrated medial and caudal to shoulder flexion (Fig. 6K). Hip flexion was concentrated medial and caudal to hip extension (Fig. 6M).

Some of the complex, multi-joint movements were more consistent across fields. Forelimb “forward/raise” movements were concentrated in more lateral portions of M1, 3a, and 3b, while forelimb retractions were concentrated in more medial and caudal portions of the same fields (Fig. S6A). This is similar to how forelimb movements are distributed in 3b vs. M1 in mice (Matyas et al. 2010) and rats (Brown and Teskey 2014; Halley et al. 2020). The relationship between cortical areas (defined histologically) and functional domains (defined experimentally) remains poorly understood, particularly for higher order fields and, as in the present study, for areas involved in motor control.

### Expanded representations of the shoulder and tongue

An important finding in the current study is that in the galago, unlike other primates studied, a relatively small region of cortex represented movements of the digits (5.3%)—a finding consistent with previous studies of galago M1 utilizing ST-ICMS (Wu et al. 2000). On the other hand, the representation of the shoulder was extremely large and assumed over a third of the entire movement map representation across all cortical fields. Like previous studies, we found regions in M1 and PPC where digit movements that were part of reaching and grasping behaviors were observed, but these were relatively small when compared to the large representation of the shoulder. Often, flexions of the shoulder (moving the whole forelimb forward) were accompanied by hip and knee extension (Fig. 7B); this synergy resembles the multi-joint movements involved in vertical leaping, a form of locomotion in galagos.

We also found an exceptionally large representation of the tongue across multiple cortical fields. This extreme magnification of the tongue is comparable to the Egyptian fruit bat (Halley et al. 2022), and in fruit bats may be an adaptation for frugivory and/or echolocation, which is accomplished by tongue clicks. This magnification of the galago tongue representation may be related to its diet of fruit, insects, and tree gum (Harcourt and Nash 1986). Tongue movement types were distributed across the cortex in a similar fashion to forelimb movements. For example, in the tongue representation in 3b, most evoked movements were retraction of the tongue into the mouth, while in M1 stimulation produced extensions of the tongue out of the mouth, including large lateral extension (e.g. Fig. 5D). Thus, the full complement of tongue movements is distributed across these cortical areas.

### Current results compared with previous studies

A number of studies have characterized the movements elicited from stimulation of M1 and premotor cortex in galagos using both short-train ICMS (Fogassi et al. 1994; Wu et al. 2000) and long-train ICMS (Cooke et al. 2015; Stepniewska et al. 2020; Wang et al. 2021). In general, the portion of our maps that cover M1 and PM agree with the findings of these studies in terms of gross topographic organization, but they also differ in some important ways. First, our maps differ from short-train studies (Wu et al. 2000; Fang et al. 2005), as we describe multi-joint movements of muscle synergies elicited at individual sites, rather than single-joint representations at minimal thresholds. Second, compared with previous LT-ICMS studies of M1 (Cooke et al. 2015; Stepniewska et al. 2020; Wang et al. 2021), we describe the directional movements of individual joints, rather than grouping them according to ethological categories.

Our study elicited movements from multiple areas of the galago PPC using LT-ICMS, and while we applied a different analytic technique, our results largely agree with the distribution of movement types described in previous reports. For example, we found that jaw opening was only elicited from stimulation of PPC areas that were lateral to the IPS (Figs. 3, 4), consistent with the suite of movements involved in the “face aggressive” grouping, relative to the “face defensive” one (Stepniewska et al. 2005; 2009; Cooke et al. 2015). We did not observe eye movements in any portion of PPC (Fig. 5H) as previously reported (Stepniewska et al. 2009).

In previous studies, complex movements of the forelimb in PPC were evoked medial to the IPS. These included “avoidance,” “defensive,” “hand to mouth,” and “reaching.” In the current study we found that in cortex medial to the IPS stimulation elicited forward movements of the shoulder (Fig. 6K), digit flexion (Fig. 6I), and forelimb adduction (Fig. S6B), which are components of the more complex movements evoked in the previous studies. However, in the present study, these forelimb movements overlapped with mouth opening only lateral to the IPS (Fig. 4G). We did not observe full forelimb retraction in any area caudal to area 3b (Fig. S6A), but our study did find a distribution of shoulder movements consistent with the rostral “forelimb defensive” vs. caudal “reaching” regions. Specifically, shoulder movements were backward in rostral portions of PPC (consistent with retraction), forward in caudal sites of PPC (consistent with reaching), and mixed in areas 1/2 (Fig. 6K).

Finally, previous studies found that the most medial areas of PPC elicited movements of the hindlimb and forelimb in tandem, with hindlimb movements largely bilateral and originating at the hip (Stepniewska et al. 2005, 2009; Wang et al. 2021). We found similar results in two cases (Fig. 3A, Fig. 4A), with several sites in this region eliciting knee, ankle, and toe flexion as well in one case (Fig. 3A). Note that in our study, these movements were elicited from areas 1/2 and caudal 3b, rather than PPC. Many combined movements of both forelimb and hindlimb are similar to limb coordination involved in running or climbing (Wang et al. 2021).

### “Intrinsic” vs. “extrinsic” movement representation in sensorimotor cortex

A central question in sensorimotor neuroscience concerns whether neurons represent (i) the activation of individual muscles, (ii) the movement of joints along anatomical axes (e.g. flexion/extension, involving multiple muscles), or (iii) extrinsic space, independent of the muscles or joint activations required for movement in a particular direction (Kakei et al. 1999, 2001). Kakei et al. (1999) designed an elegant experiment to distinguish between these possibilities, comparing neuron coactivation with “intrinsic” movements (either muscle activations or joint movements) and “extrinsic” movements (directions in external space). They recorded from individual M1 neurons in awake behaving macaques, and found evidence that the hand region of M1 contained neurons whose firing covaried with both wrist muscle activation (“intrinsic”) and directional wrist movements (“extrinsic”), but not with joint movements.

The current study found that joint movements in the galago differ systematically across the sensorimotor cortex, consistent with previous studies using identical methods in rats (Halley et al. 2020) and fruit bats (Halley et al. 2022), as well as findings in mice (Matyas et al. 2010). Methodological differences make it difficult to interpret our results relative to Kakei et al. (1999). We stimulated populations of neurons in anesthetized animals, rather than recording from single neurons in awake animals, and our experimental setup did not allow us to distinguish between movements that are “intrinsic” (muscular or joint) and “extrinsic” (directions in space). However, the distribution of joint movement types across cortical fields (e.g. tongue [Fig. 5b], forelimb [Fig. 6I-L], hindlimb [Fig. 6M-N]) suggests that parietal areas of cortex play a distinct role from M1 in motor action, similar to the “intrinsic” and “extrinsic” neuron subpopulations in M1 described previously (Kakei et al. 1999).

### The evolution of motor cortex in mammals and networks associated with reaching and grasping

This study is part of a larger comparative effort in our laboratory to characterize areas of the neocortex involved in motor control utilizing similar intracortical microstimulation techniques and methods of analysis. The goal is to leverage the method of LT-ICMS in order to distinguish movement types that are difficult to study using ST-ICMS (e.g. flexion vs. extension of individual joints) as well as to characterize movement synergies that may be unique to individual species. Galagos and other prosimian primates are thought to most closely resemble the last common ancestor of all primates (Charles-Dominique and Martin 1970; Silcox et al. 2009; Steiper and Seiffert 2012), especially in the organization of their neocortex (Kaas 2012). Therefore, they represent an essential link to translating results from rodent species that serve as model organisms in neurobiology to primates, including humans.

An important finding in the current study is the paucity of stimulation sites in any cortical field examined in which movements of the digits alone were evoked. A lack of distinct digit representations was also found in previous studies of M1 utilizing ST-ICMS in galagos (Wu et al. 2000). This result indicates that early primates likely had relatively simple networks for reaching and grasping that do not appear to be associated with fine control of the digits per se, but rather with grasping using all digits, as well as vertical leaping (see below). This is starkly different from studies in both New and Old World monkeys in which a large swath of cortex is devoted to the representation of digit movements (Baldwin et al. 2017b; Baldwin et al. 2018; Mayer et al. 2019). Specifically, movements evoked in monkeys were associated with multiple types of precision grips (macaques and capuchins) or power grasps (titi monkeys).

Our comparative work also indicates that cortical networks for reaching and grasping are present in non-primate mammals such as rats and tree shrews (Baldwin et al. 2017a; Halley et al. 2020). For example, in rats M1 and area 3b are involved in motor control of the limbs, but studies using LT-ICMS found no regions in which the digits alone are represented (Halley et al. 2020). However, individual digit movements are observed in the rostral forelimb area in studies using ST-ICMS (Brown and Teskey 2014). In tree shrews, one of the closest living relatives to primates, motor control of the limbs is distributed across M1, 3b, and PPC, but there are only a few sites in which stimulation elicits movements of the digits alone. Taken together, the data indicate that rudimentary cortical networks for reaching and grasping in non-primate mammals expanded in early primates, but still did not include regions that were involved in fine motor control of the digits. These manual abilities and the cortical areas that generate them evolved later in primate evolution, and changed dramatically, likely due to the evolution of an opposable thumb in some lineages and the sophisticated dexterous behaviors that are the hallmark of our own species’ evolution.

## Supplementary Material

0_Galago_ICMS_Supp_v19_bhaf195
