# Supplementary material for: Movement representations in motor, somatosensory, and posterior parietal cortex of the greater galago
Source: Cereb Cortex. 2025 Aug 21;35(8):bhaf195. doi: 10.1093/cercor/bhaf195 (PMC12368956; doi:10.1093/cercor/bhaf195)
Supplement: 0_Galago_ICMS_Supp_v19_bhaf195 [file 0_galago_icms_supp_v19_bhaf195.docx]

**Supplementary Materials**

**Title: Movement representations in motor, somatosensory, and posterior parietal cortex of the greater galago**

**Authors:** Andrew C. Halley^1^, Iwona Stepniewska^2^, Qimeng Wang^2^, Jamie L. Reed^3^, Hui-Xin Qi^2^, Jon H. Kaas^2^, Leah A. Krubitzer^1,4^

1. Center for Neuroscience, University of California, Davis, Davis, CA, USA.
2. Psychological Sciences, Vanderbilt University, Nashville, TN, USA.
3. Department of Radiology, Vanderbilt University, Nashville, TN, USA.
4. Department of Psychology, University of California, Davis, Davis, CA, USA.

**Corresponding Author:**

Leah Krubitzer

Krubitzer Laboratory
University of California, Davis
Center for Neuroscience
1544 Newton Court
Davis, CA 95618

Phone: (530) 757-8868

Fax: (530) 757-8827

Email: lakrubitzer@ucdavis.edu

Running Title: The evolution of primate motor control

**
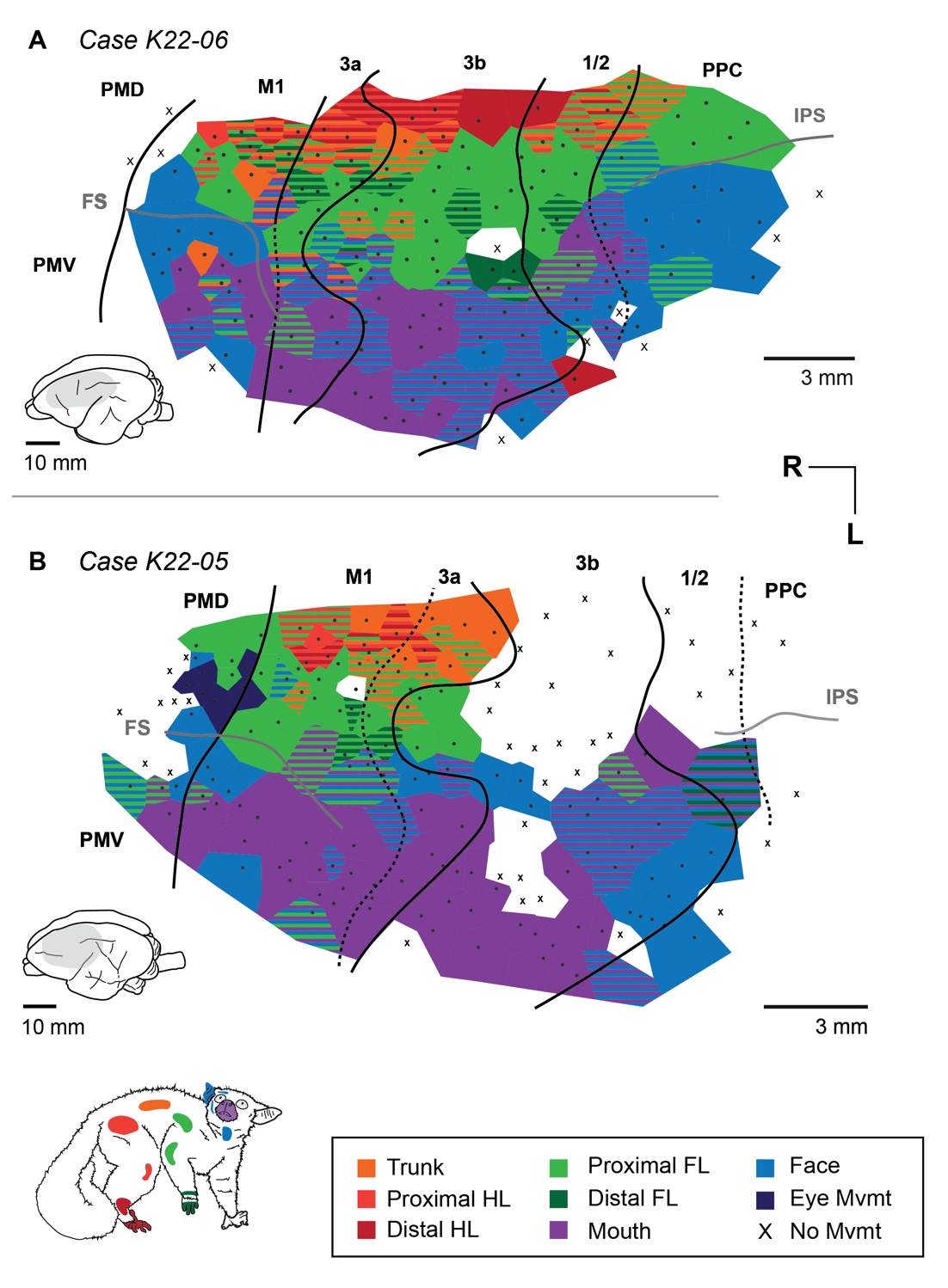
**

**Figure S1.** Simplified motor maps of the two cases presented in Figure 3. Conventions follow those in Figure 3, with anatomical movements grouped more broadly. “Distal HL” includes toes and ankle. “Proximal HL” includes knee and hip. “Distal FL” includes digits and wrist. “Proximal FL” includes elbow and shoulder. “Mouth” includes jaw, tongue and lip. “Face” includes neck, snout, ear, and eye blinks.

**
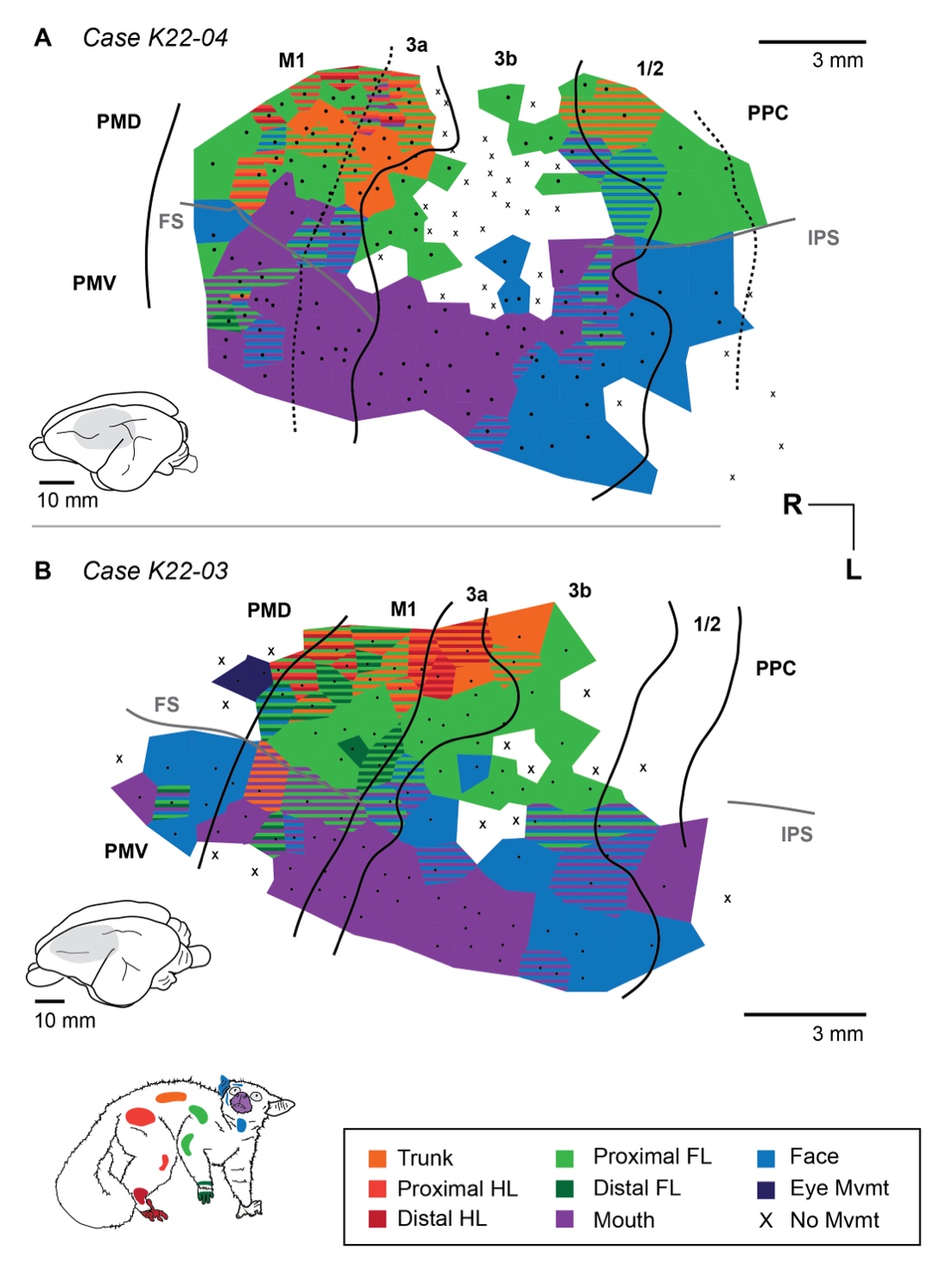
**

**Figure S2.** Simplified motor maps of the two cases presented in Figure 4. Conventions follow those in Figure 4, with anatomical movements grouped more broadly. “Distal HL” includes toes and ankle. “Proximal HL” includes knee and hip. “Distal FL” includes digits and wrist. “Proximal FL” includes elbow and shoulder. “Mouth” includes jaw, tongue and lip. “Face” includes neck, snout, ear, and eye blinks.


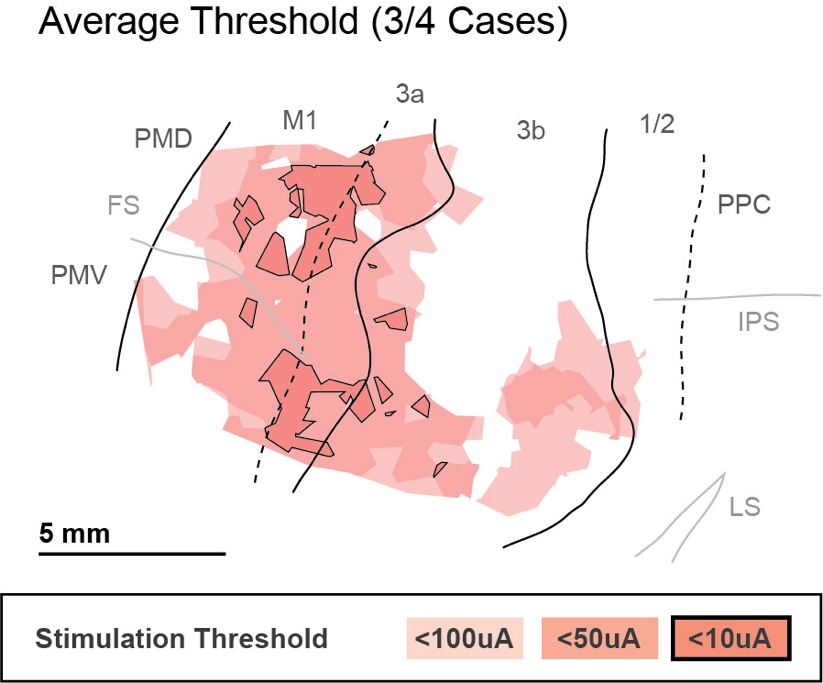


**Figure S3.** Long-train intracortical microstimulation (LT-ICMS) thresholds averaged across four cases of the galago. Areas of especially low threshold stimulation include M1 and 3a, as well as lateral portions of 3b.

**
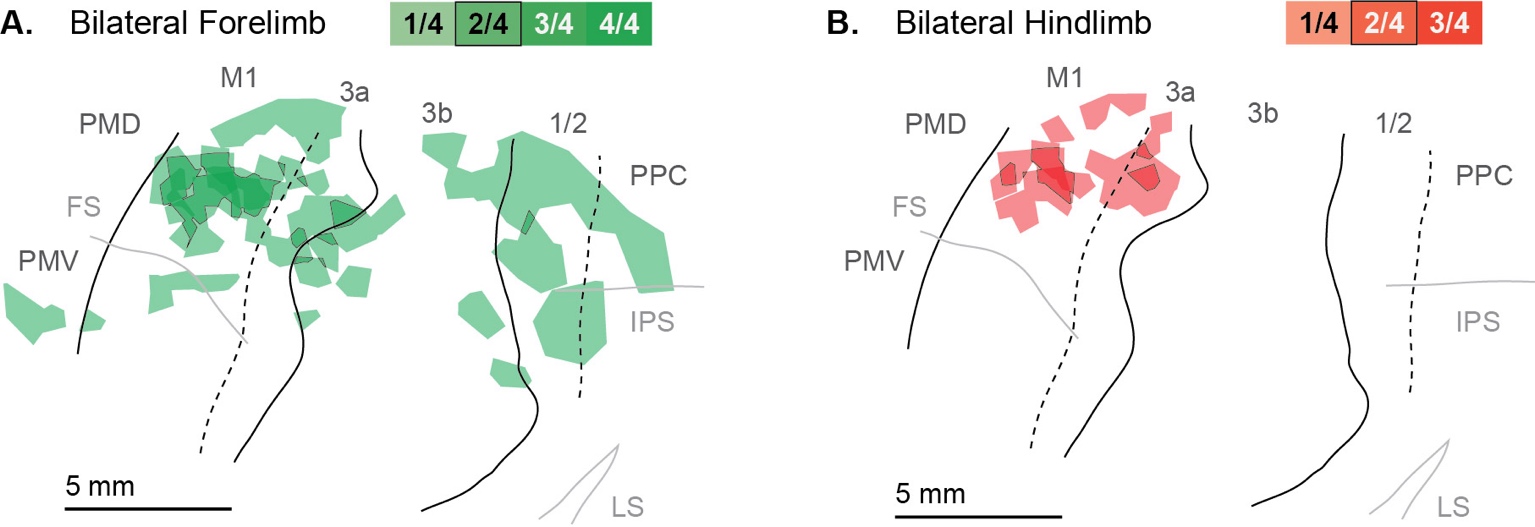
**

**Figure S4**. Bilateral movements of the limbs elicited from stimulation across cases. (A) Bilateral forelimb movements were concentrated in rostral portions of 3b, 3a, M1, and adjacent motor fields. (B) Bilateral hindlimb movements were primarily elicited from areas 3a and M1


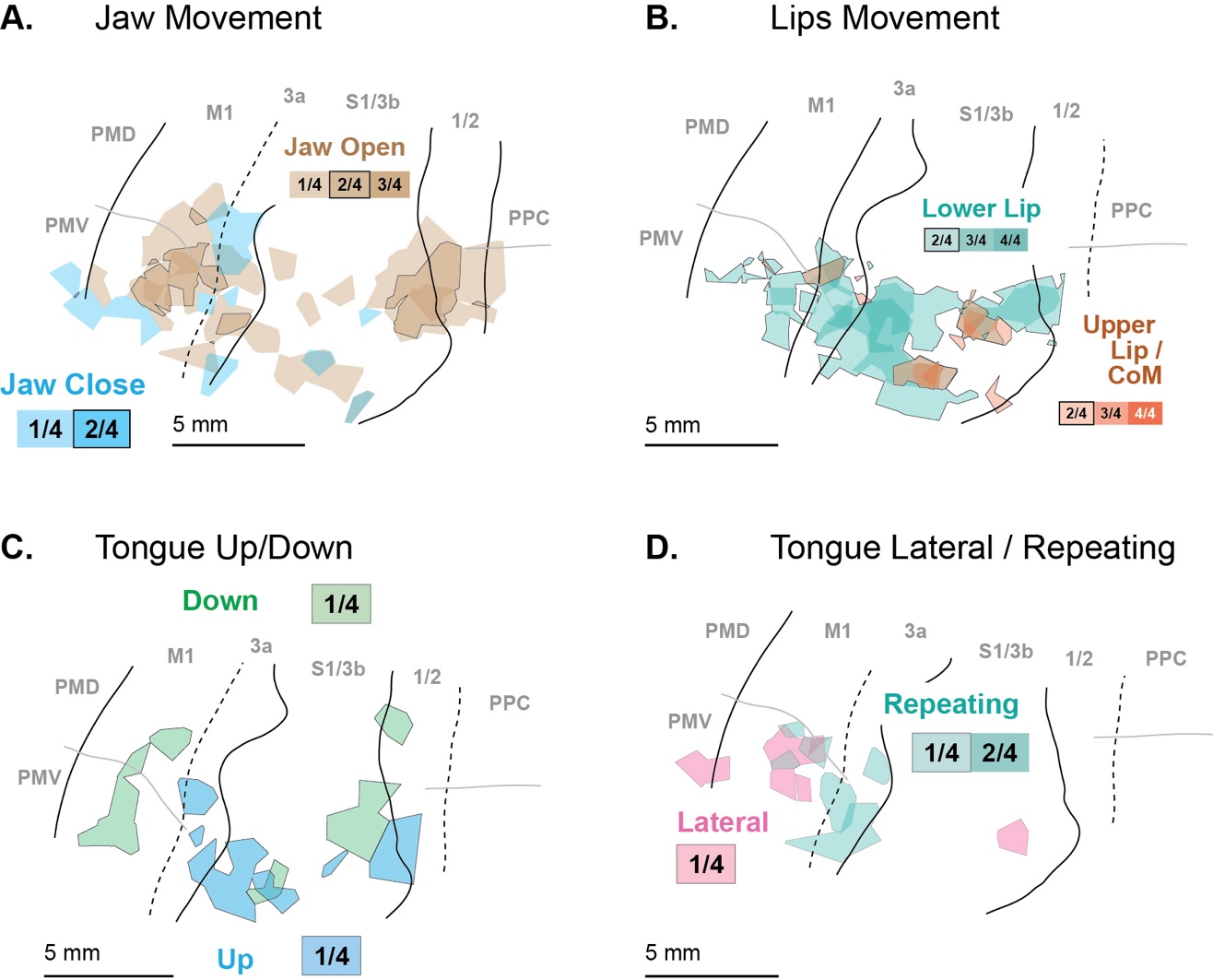


**Figure S5.** Detailed movements of the jaw, lips, and tongue across cases. (A) Two areas elicited widespread closing of the jaw (comprising most jaw movements), one in a rostrolateral region spanning rostral 3b, 3a, and M1, and another along the caudal boundary of S1 and portions of areas 1 and 2. Jaw closures were scattered but most common adjacent to the rostral area. (B) Lower lip movements were widespread and formed a near-continuous region spanning M1, 3a, S1, and areas 1/2. Movements of the upper lip and corners of the mouth (CoM) were elicited in medial portions of the lip area of 3a, as well as a region of caudolateral S1. (C) Localization and upward vs. downward tongue movements. (D) Lateral tongue movements were concentrated in M1, and repetitive tongue movements across the stimulation period were concentrated in rostral S1, 3a, and M1.

**
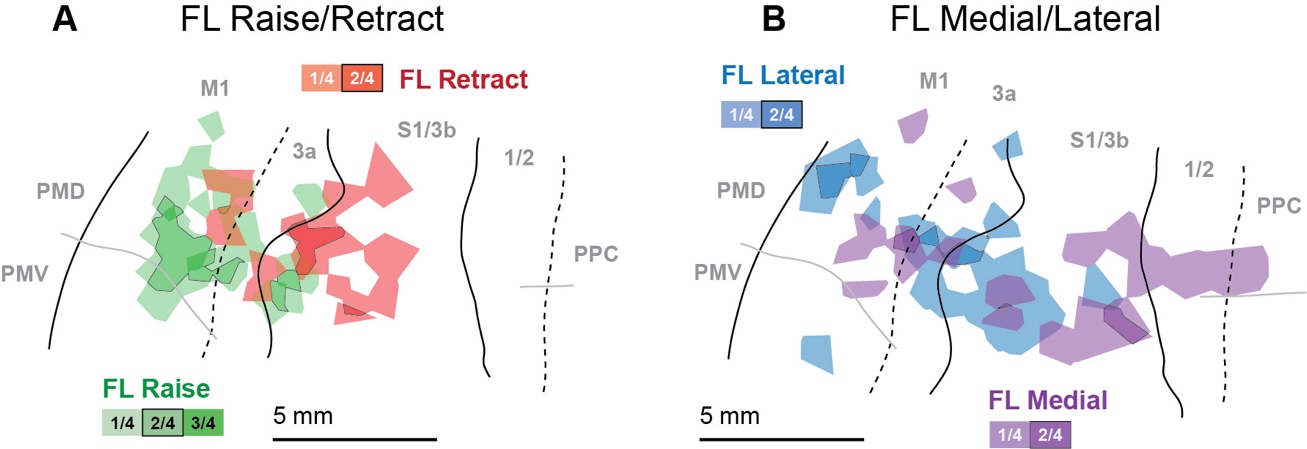
**

**Figure S6**. Forelimb movement types observed across all cases. (A) Forelimb movements involving multiple body parts in a forward-raise vs. retraction direction. Forward movements were concentrated in rostral S1/3b, 3a, and M1. Retraction movements were concentrated in more medial portions of S1, with some retractions in 3a and M1. (B) Forelimb movements that moved the limb medially vs. laterally were observed across most cortical fields. Additional directional movements of the forelimb are shown in Fig. 6K (shoulder flexion and extension) and Fig. 6I (forelimb digit flexion and extension).

**
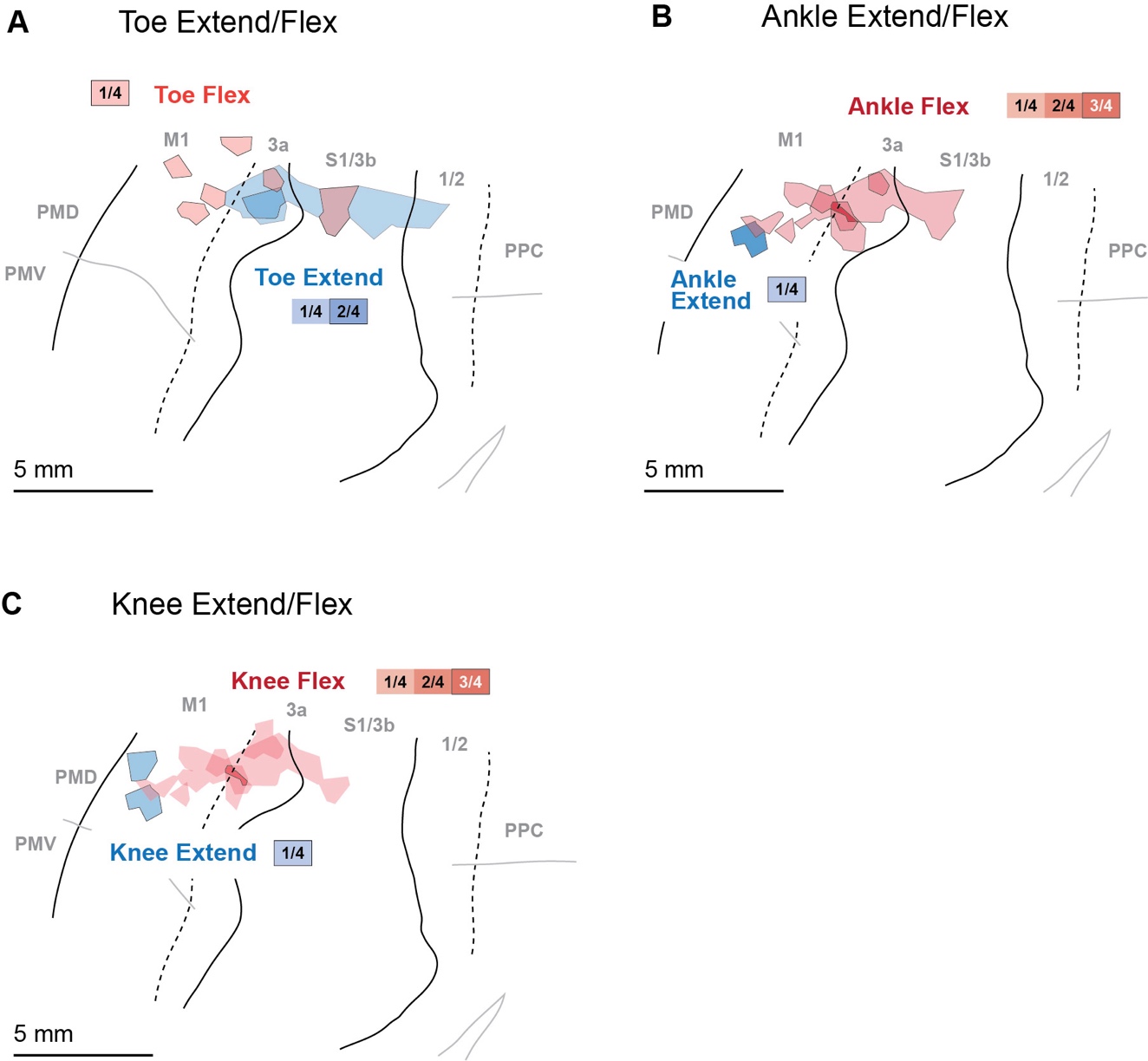
**

**Figure S7.** Hindlimb movement types observed across all cases. (A) Toe extensions were observed primarily in S1/3b and 3a, while toe flexions were observed in M1, 3a, and S1. (B) Ankle flexions were observed in S1, 3a, and M1. Ankle extensions were rare, and only observed in more lateral portions of the M1 ankle representation. (C) Knee flexions were observed in S1, 3a, and M1, while knee extensions were less common and only observed along the rostral border of M1. Directional hip movement types are described in Fig. 6M.
